# Genome-Wide Identification and Expression Profiling of Potassium Transport-Related Genes in *Vigna radiata* under Abiotic Stresses

**DOI:** 10.3390/plants11010002

**Published:** 2021-12-21

**Authors:** Farrukh Azeem, Usman Ijaz, Muhammad Amjad Ali, Sabir Hussain, Muhammad Zubair, Hamid Manzoor, Muhammad Abid, Roshan Zameer, Dong-Seon Kim, Kirill S. Golokhvast, Gyuhwa Chung, Sangmi Sun, Muhammad Amjad Nawaz

**Affiliations:** 1 Department of Bioinformatics and Biotechnology, GC University, Faisalabad 38000, Pakistan; azeuaf@hotmail.com (F.A.); usmanijazahmad1246@gmail.com (U.I.); muhammad.zubair1751@gmail.com (M.Z.); roshanzameer23@gcuf.edu.pk (R.Z.); 2 Department of Plant Pathology, University of Agriculture, Faisalabad 38000, Pakistan; amjad.ali@uaf.edu.pk; 3 Department of Environmental Science and Engineering, GC University, Faisalabad 38000, Pakistan; sabirghani@gmail.com; 4 Institute of Molecular Biology & Biotechnology, Bahauddin Zakariya University, Multan 60800, Pakistan; hamid_1249@yahoo.co.uk; 5 Department of Plant Pathology, Bahauddin Zakariya University, Multan 60800, Pakistan; abiduvb@gmail.com; 6 KM Research Science Division, Korea Institute of Oriental Medicine (KIOM), Daejeon 34054, Korea; dskim@kiom.re.kr; 7 N.I. Vavilov All-Russian Research Institute of Plant Genetic Resources, 190000 Saint Petersburg, Russia; droopy@mail.ru; 8 SEC in Nanotechnology, Engineering School, Far Eastern Federal University, 690922 Vladivostok, Russia; 9 Siberian Federal Scientific Center of Agrobiotechnology, Russian Academy of Sciences, Krasnoobsk, 630501 Novosibirsk, Russia; 10 Department of Biotechnology, Chonnam National University, Yeosu Campus, Gwangju 52626, Korea; chung@chonnam.ac.kr; 11 Laboratory of Supercritical Fluid Research and Application in Agrobiotechnology, The National Research Tomsk State University, 36, Lenin Avenue, 634050 Tomsk, Russia

**Keywords:** K^+^ transporters, K^+^ channels, drought stress, heat stress, salt stress, RNA-seq, KUP/HAK/KT, KEA, Shaker family, TPK

## Abstract

Potassium (K^+^) is one of the most important cations that plays a significant role in plants and constitutes up to 10% of plants’ dry weight. Plants exhibit complex systems of transporters and channels for the distribution of K^+^ from soil to numerous parts of plants. In this study, we have identified 39 genes encoding putative K^+^ transport-related genes in *Vigna radiata*. Chromosomal mapping of these genes indicated an uneven distribution across eight out of 11 chromosomes. Comparative phylogenetic analysis of different plant species, i.e., *V. radiata*, *Glycine max*, *Cicer arietinum*, *Oryza sativa*, and *Arabidopsis thaliana,* showed their strong conservation in different plant species. Evolutionary analysis of these genes suggests that gene duplication is a major route of expansion for this family in *V. radiata*. Comprehensive promoter analysis identified several abiotic stresses related to cis-elements in the promoter regions of these genes, suggesting their role in abiotic stress tolerance. Our additional analyses indicated that abiotic stresses adversely affected the chlorophyll concentration, carotenoids, catalase, total soluble protein concentration, and the activities of superoxide and peroxidase in *V. radiata*. It also disturbs the ionic balance by decreasing the uptake of K^+^ content and increasing the uptake of Na^+^. Expression analysis from high-throughput sequencing data and quantitative real-time PCR experiments revealed that several K^+^ transport genes were expressed in different tissues (seed, flower, and pod) and in abiotic stress-responsive manners. A highly significant variation of expression was observed for *VrHKT* (1.1 and 1.2), *VrKAT* (1 and 2) *VrAKT1.1*, *VrAKT2*, *VrSKOR*, *VrKEA5*, *VrTPK3*, and *VrKUP/HAK/KT* (4, 5, and 8.1) in response to drought, heat or salinity stress. It reflected their potential roles in plant growth, development, or stress adaptations. The present study gives an in-depth understanding of K^+^ transport system genes in *V. radiata* and will serve as a basis for a functional analysis of these genes.

## 1. Introduction

The potassium ion (K^+^) is an important inorganic macro-nutrient for plant growth and the fourth abundant mineral in the lithosphere. K^+^ constitutes up to 10% of the total plant dry weight [1]. Plants utilize K^+^ for several important processes like osmoregulation, cell elongation, the control of membrane polarization, and the electrical neutralization of anionic groups. This ion also helps in maintaining the pH of the cytosol, which is crucial for the proper functioning of most enzymes. Within the cell, it is present in the nucleus, vacuoles, and chloroplast [2,3]. K^+^ is also important for maintaining the K^+^ concentration in the cytosol. Despite its abundance and importance in the cell, the optimal concentration of K^+^ is sustained for the proper functioning of the cell. A plant’s ability to tolerate drought [4] and salinity stress [5] has also been associated with the maintenance of K^+^ concentration in the cytoplasm. In plants, K^+^ is abundantly present in the cytosol (i.e., 60–160 mM) and its concentration is regulated by a complex transport system involving channels and transporters [6]. This transport system facilitates the absorption of K^+^ from the soil and carries it into the plant body. In *Arabidopsis thaliana*, 35 K^+^ transport-related genes (including 20 transporters and 15 channels) have been predicted [7,8].

In plants, numerous proteins (both channels and transporters) mediate cellular K^+^ uptake and distribution. The channel proteins for K^+^ transport belong to voltage-dependent Shaker-like channels, voltage-independent tandem-pore K^+^ (TPK) channels, and two-pore channels (TPC) [9]. The Shaker family is further divided into five subgroups: weak inward rectifying channels, KAT-like inward rectifying channels, AKT-like inward rectifying channels, outward rectifying channels, and the silent/regulatory subunit [10]. Moreover, the carrier-like KT/HAK/KUP family [11,12], HKT uniporters and symporters [12], and KEA antiporters [13] are also involved in this process. The KUP/HAK/KT family is responsible for high-affinity potassium uptake [12]. The HKT proteins are important for the uptake and homeostasis of Na^+^ and K^+^. In plants, there are two classes of HKTs (HKT1-like and HKT 2-like). Class I HKTs are Na^+^ uniporters and class II HKTs are Na^+^ and K^+^ symporters [14]. The KEA proteins are closely related to bacterial KefC K^+^/H^+^ antiporters [15,16]. Most of the KEAs are expressed at the chloroplast membranes and regulate the pH of thylakoids and stroma [17]. KEA 4, 5, and 6 take part in maintaining pH and K^+^ homeostasis in endomembrane compartments [18,19]. The K^+^ channels are multimeric proteins comprising trans-membrane segments and are quantified based on pore domains (PD). Four PDs are linked with functional multimeric proteins to make a conduction pathway of channels. A highly conserved motif, i.e., “GYGD/E” is present in the P domain of the K^+^ channel. Based on the topology of K^+^ channels, 15 K^+^-selective channels are classified into three families in *A. thaliana*, which include one K^+^ inward rectifier (Kir-like), nine voltage-gated ion channels, and five tandem-pore K^+^ channels (TPK). K^+^ transporters are also grouped into three families including the KEA (K^+^/H^+^ antiprotons) family of K^+^ efflux antiporters (6 members), the KUP/HAK/KT family of K^+^ uptake permeases (13 members), and the Trk/HKT family of high-affinity K^+^ transporters (1 member) [20].

Mung bean (*Vigna radiata*) is an important diploid pulse crop (2n = 2x = 22), largely cultivated in tropical and subtropical regions. The seeds of mung beans are an excellent source of carbohydrates, fats, proteins, and micronutrients [21]. The production of mung beans has been increased annually, mostly in Asian countries, i.e., Bangladesh, India, and Pakistan. Adaptation to extreme environmental conditions, supported by evolution, makes *V. radiata* a rich pool of genes related to stress tolerance [22,23]. The increasing availability of genomic and transcriptomic resources has provided an opportunity to conduct an evolutionary and comparative analysis of various gene families in *V. radiata*. Owing to the important role of K^+^ in various physiological processes, as well as abiotic and biotic stress tolerances, in *V. radiata* [24], it is essential to delineate the K^+^ transporter compendium within it.

Various K^+^ transporters and channels are well-categorized in legumes, e.g., *Cicer arietinum* [25], or *Glycine max* [26], but there is no information available on K^+^ transporters and channels in mung beans (*V. radiata*). The current study was planned to identify the K^+^ channels and transporters in *V. radiata*.

## 2. Results

### 2.1. Identification and Phylogenetics of K^+^ Transporters and Channels

**Figure 1:**
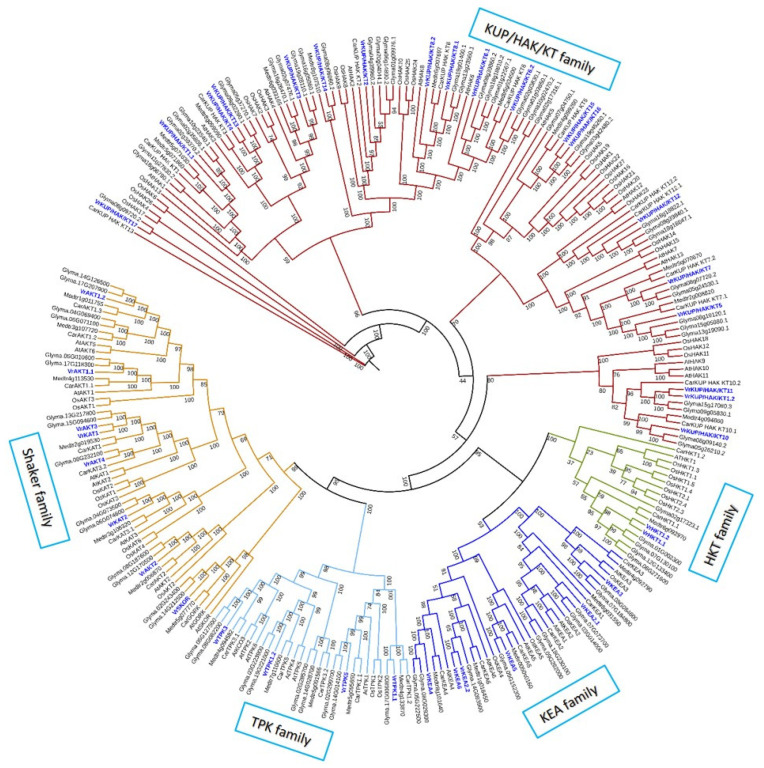
Neighbor-joining (NJ) tree of K^+^ transporters and channels in *V. radiata* and related species. The evolutionary history was inferred from protein sequences by the neighbor-joining method, based on a JTT model. The tree with the highest log likelihood (-119897.9497) is shown. Initial tree(s) for the heuristic search were obtained automatically by applying neighbor-join and BioNJ algorithms to a matrix of pairwise distances estimated using a JTT model, and then selecting the topology with a superior log-likelihood value. Evolutionary analyses were conducted in MEGA7 [35]. The tree was visualized and edited in iTOL [36].

After confirming the conserved domains and motifs, and carefully surveying the *V. radiata* genome, 39 putative K^+^ transport genes were identified in *V. radiata* (Figure 1, Table 1). This number is higher than in *A. thaliana* (35 members) and lower than in *Oryza sativa* (68 members) [7,27]. The genome sizes of *A. thaliana*, *O. sativa,* and *V. radiata* are almost 135, 370, and 460 Mbs, respectively. It predicts that the number of K^+^ transport-related genes is not associated with genome size. K^+^ transport genes were classified into 12 K^+^ channels and 27 K^+^ transporters. The average molecular weight of the identified K^+^ transport proteins ranged from 38.65153 to 94.30594 KDa, with the isoelectric point from 4.55 to 9.39 (Table 1). In *V. radiata*, 34 out of 39 genes were mapped on eight chromosomes, while five genes (VrKUP/HAK/KT12, VrKUP/HAK/KT6.1, VrKUP/HAK/KT6.2, VrKUP/HAK/KT3, and VrAKT4) were present on the scaffold regions (Table 1). 

The neighbor-joining (NJ) tree (Figure 1) showed that the K^+^ transporters and channels formed distinct familywise clades, i.e., K^+^ transporters (KUP/HAK/KT, HKT, KEA) and channels (Shaker and TPK).

#### 2.1.1. The K^+^ Transporters in *V. radiata*

In the *V. radiata* genome, 19 KUP/HAK/KT members were identified that exhibited the same pattern of the domain as their counterpart in *A. thaliana*. The number of genes is comparatively smaller than *O. sativa* (25 members) [28], *Triticum* *aestivum* (56 members) [29], and greater as compared to *A. thaliana* (13 members) and *C. arietinum* (15 members) [25]. The length of the predicted VrKUP/HAK/KT proteins ranged from 723 (VrKUP/HAK/KT17) to 846 (VrKUP/HAK/KT7) amino acids (Table 1). The frequency of transmembrane segments (TMS) ranged from 10 to 14, which is quite similar to that of KUP/HAK/KT members in *A. thaliana* and *O. sativa*, i.e., 11–15 [30]. VrKUP/HAK/KTs contain 14 TMS and another K^+^ transporter domain (K_trans) (Table 1, Figure S1). This additional trans-domain is involved in the transport of K^+^. Most of the members of this family are high-affinity transporters [31].

Two members of the Trk/HKT (VrHKT1.1 and VrHKT1.2) family were also identified, which are 507 and 518 amino acids long, respectively. The members of the HKT family help to transport K^+^/ Na^+^ and possess a similar topology as K^+^ channels. Members of the HKT family comprise four P-loops and eight TM domains. HKT family members are divided into subgroups, based on the presence of serine (S) or glycine (G) residues. In the HKTs of subfamily I, a conserved S residue is present at the end of the first P-loop (MPAM motif). Subfamily II members contain G residue instead of S residue at the end of the first P-loop. The presence of an S or G amino acid in the MPAM motif regulates the K^+^ or Na^+^ permeability. Plant HKTs act as a Na^+^-K^+^ symporter when glycine (Gly) exists in the MPAM motif. However, HKT transporters merely show Na^+^-selective permeability when Gly is substituted by serine (Ser). Therefore, plant HKTs can be classified according to SerGlyGlyGly-type (subfamily I) and GlyGlyGlyGly-type (subfamily II). AtHKT1.1 belongs to subfamily I because it contains S residue and is involved in the transport of Na^+^. In *C. arietinum,* two members (CarHKT1.1 and CarHKT1.2) belong to subfamily I and are potentially involved in Na^+^ transport [25]. In *V. radiata*, there is a conserved “S” residue in both proteins (VrHKT1.1 and VrHKT1.2) at positions 72 and 82, respectively. Therefore, both VrHKTs belong to subfamily I and are potentially involved in Na^+^ transport.

Since HKTs are known to have glycosylation sites (NXS/T, where X presents for any amino acid) [32,33] we, therefore, found three and two N- glycosylation sites in VrHKT1.1 (at positions 2-4,122-124 and 133-135) and VrHKT1.2 (at positions 7-9 and 169-171), respectively.

In *A. thaliana,* six KEA members have been reported, consisting of H^+^/K^+^ antiporters [34]. We found six members of the KEA family (named VrKEA2.1, VrKEA2.2, VrKEA3, VrKEA4, VrKEA5, and VrKEA6). The average number of introns present in the KEA family is nine. Multiple sequence alignment specifies that the “G” residue is conserved at various positions between the members of *V. radiata* and *A. thaliana*.

#### 2.1.2. K^+^ Channels in the *V. radiata* Genome

The name “Shaker” originates from the initial member (the Drosophila Shaker (Sh)) of the family, first cloned in *Drosophila* [37]. K^+^ channels comprise the cNMP (cyclic nucleotide-binding) domain, present at the cytosolic C- terminal, and the KAH domain, richly hydrophobic and acidic. The ANK domain is also present between cNMP and KAH and interrelates with regulatory proteins. Eight Shaker genes were found in *V. radiata* (one SKOR, two AKT1s, one AKT2, one AKT3, one AKT4, one KAT1, and one KAT3) in contrast to *A. thaliana* (9), *Vitis vinifera* (9), and *O. sativa* (11) [10,27,38] (Table 1). These genes had domain patterns similar to the ones present in *Arabidopsis,* i.e., ANK, Ion_trans_2, and KHA [39]. Shaker channels are divided into five groups, i.e., the AKT1 channel, silent channel, KAT1-like inward channels, weak outward channels, and weak inward rectifying channels [25,31]. The peptide length ranged from 623 to 869, while the genes contained six to nine introns. In *V. radiata,* all members of the Shaker family are considered as K^+^-selective channels because the “TxxTxGYGD” motif is conserved among them [40]. In the phylogenetic tree, the Shaker family appears as a separate group.

**Figure 2:**
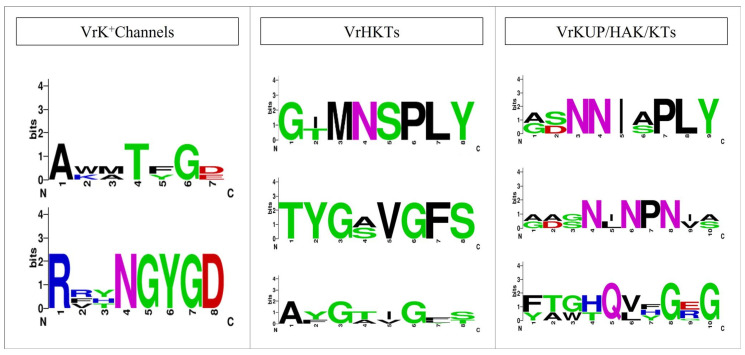
Conserved motifs present in the alignment of K^+^ channels and the transporters’ protein sequences from *V. radiata*, *O. sativa* and *A. thaliana*. The overall height of each column indicates conservation at that position in the alignment, whereas the height of each letter within the column indicates the relative frequency of each amino acid at that position.

The TPK family is also recognized in other plant species, i.e., *A. thaliana*, *Solanum tuberosum*, *O. sativa*, *Hordeum vulgare,* and *Nicotiana tabacum* [41,42,43]. Initially, Kir-like channels were considered as a distinct group, while they show a great similarity with the TPK family. Plant Kir-like channels are discovered only in the Arabidopsis genus [44]. Four members of TPK were identified in *V. radiata*, while one member of the Kir-like family was identified. A highly conserved motif “RSXpS/pTXP” was found at the end of the N-terminal. All four members of TPK have the same topology (5TM/2P) as their counterparts in *O. sativa* and *A. thaliana*, i.e., the presence of GYGD and EF motifs. The GYGD motif acts as a marker of the K^+^ channel and is conserved among all members in *V. radiata*. TPK family members comprise four TM domains, a hydrophobic core, and two P-loops (also termed KCO-2P). The N-terminal analysis shows that threonine/serine is conserved in all members (Figure 2). The VrTPK peptides were 344 to 425 amino acids long. These genes contained four to six introns.

### 2.2. Gene Structure and Gene Duplications of K^+^ Transport-Related Genes in V. radiata

Exon and intron structures are more or less conserved in paralogs, while their positions in orthologs are mostly well-conserved. To investigate the diversity in gene structure among K^+^ transporters and channels in *V. radiata*, the intron-exon position was studied. There was substantial diversity in the number (2–23) and length of exons in K^+^ channels and transporters (Supplementary Figure S1). In the case of the Shaker family, *VrAKT1.1, VrAKT1.2, VrAKT2,* and *VrAKT3*, the pattern of introns/exons is the same, except for the number of introns, which is comparatively more in *VrAKT1.2*. At a genomic level, the number of exons in *VrHAK/KUP/KTs* varied from 8 to 13 (Figure S1). In addition, conserved exon analysis indicated the conservation of exons among families. In the *VrKUP/HAK/KT* family, exons 4 and 5 are conserved, and exon 2 is conserved in *VrHKT*, whereas exon 5 is conserved in the KEA family and is also conserved in AKT-like members. In addition, exon 1 is conserved in TPKs, exon 7 is conserved in *VrKAT1*, and exon 8 is conserved in *VrKAT2* (Supplementary Figure S2). 

Five tandemly and two segmentally duplicated K^+^ transport-related genes were found in the *V. radiata* genome. *VrKUP/HAK/KT1.1*/*VrKUP/HAK/KT1.2*, *VrKUP/HAK/KT8.1*/*VrKUPHAK8.2*, *VrKEA2.1*/*VrKEA2.2*, and *VrKEA5*/*VrKEA6* were tandemly duplicated. Similarly, *VrKUP/HAK/KT15* and *VrKUP/HAK/KT16* were segmentally duplicated. *VrKUP/HAK/KT10* and *VrKUP/HAK.KT17* were duplicated segmentally 35.67 mya. *VrKUP/HAK/KT1.1* and *VrKUP/HAK/KT1.2* possibly emerged 41.12 mya, as a result of gene duplication, and appears orthologous with *C. areitinum*. The gene pairs *VrKEA2.1* and *VrKEA3*, and *VrKEA5* and *VrKEA6,* are paralogs and were duplicated 38.19, and 32.18 mya, respectively. 

**Figure 3:**
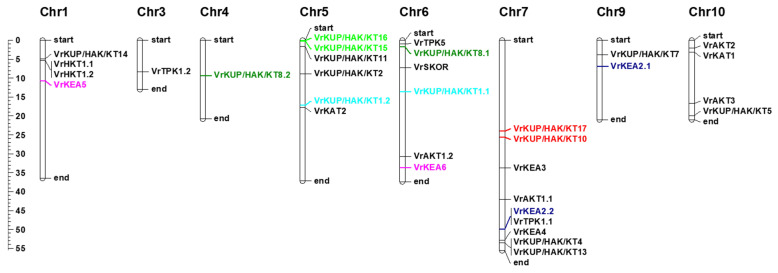
Chromosomal mapping of potassium transport-related genes. Tandem duplications are indicated by multiple colors and segmental duplications are shown in a red color. The same color indicates that the gene pair is duplicated. The scale at the left side of the chromosomal bar denotes the position on the chromosome (megabase pairs; Mb).

Among K^+^ channels, four genes, i.e., *VrAKT1.1, VrAKT1.2, VrAKT2* and *VrAKT3,* displayed a close relationship with *G. max*, *C. areitinum,* and *A. thaliana*, while two genes, i.e., *VrAKT2* and *VrAKT1.2,* exhibited an orthologous relationship with G. max. *VrKAT1* and *VrKAT3* appear as paralogs of *V. radiata* and orthologs of *G. max* KATs. The *VrSKOR* showed a close relationship with SKOR in *A. thaliana*. *VrKEA2.1* and *VrKEA3* show a paralogous relationship in *V. radiata*. Two members of the VrTPK family, i.e., *VrtTPK3* and *VrTPK5,* were duplicated 56.78 mya. Only two members of the HKTs subgroup were found (*VrAKT1.1* and *VrAKT1.2*) in *V. radiata*; both were duplicated via tandem duplication 16.53 mya. Both members show an orthologous relationship with *G. max*. Together, these results indicate that segmental and tandem duplications played a role in the expansion of K^+^ transporters and channels in the *V. radiata* genome (Figure 3).

### 2.3. Promoter Analysis of Putative V. radiata K^+^ Transporter and Channels

Cis-regulatory elements are linear fragments of non-coding DNA. Cis-regulatory elements provide the binding sites for transcription factors [45]. They have many localizations, orientations, and activities in relation to genes. Analysis of the promoter region for the cis-regulatory elements can give information about the regulatory networks of a gene. The upstream region (1000 bp upstream ATG of coding sequence) of the promoter was screened to understand the tissue-specific and functional regulation of K^+^ in *V. radiata*. Several cis-elements were found in the promoter sequence of both K^+^ channels (Table S1) and transporters (Table S2). Subsequently, in model plant species, members of HKTs and the Shaker family are well-characterized. The identified cis-elements are mainly involved in the abiotic stress response, i.e., ABRE, ACE, and the recognition site, W-box, G motif. Moreover, cis-elements involved in K^+^ transport and the light signal were also identified (Table 2). The ABA binding factor (ABF) and ABA-responsive element (AREB) are the main transcription factors activated in the abiotic stress response. Cis-regulatory elements, which have been predicted in the promoter region of K^+^ transport-related genes and the homeostasis of K^+^, are significant for stress tolerance.

### 2.4. Physiological and Biochemical Response of V. radiata to Drought, Heat, and Salt Stresses

**Figure 4:**
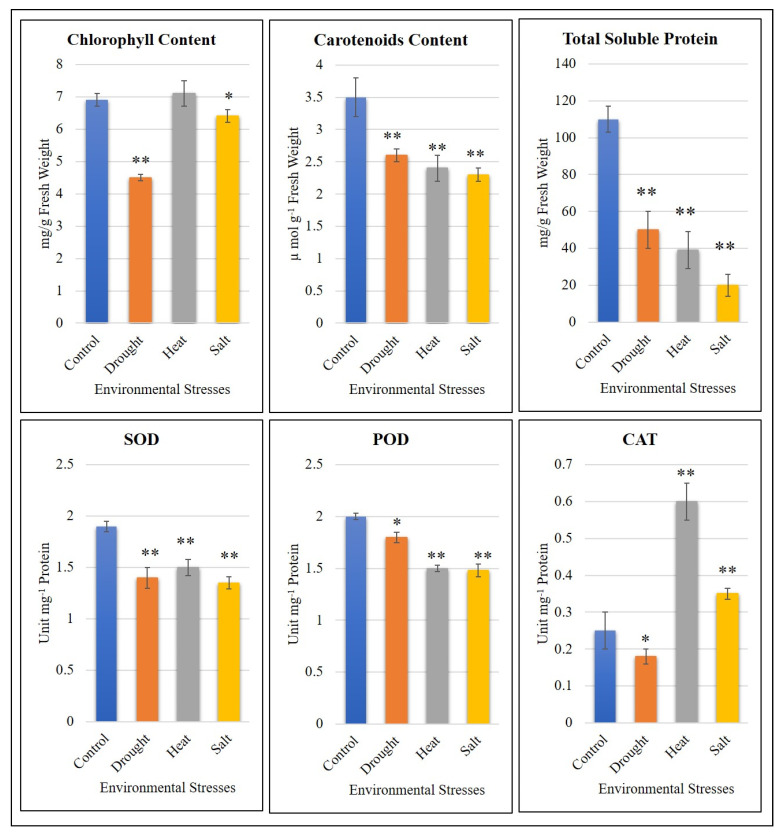
Effect of salinity, drought, and heat stress on carotenoids, chlorophyll, total soluble protein concentration, catalase activity (CAT), peroxidase activity (POD), and superoxide activity (SOD). One unit of enzyme activity represents the amount of enzyme that breaks down 1 µmol of H_2_O_2_/min under the assay conditions. The asterisk sign indicates significant changes compared to control, salt, heat, and drought stress (* *p* < 0.05, ** *p* < 0.01).

According to our results, the total chlorophyll concentration was adversely affected by drought stress compared to salt stress, while there was no significant effect from heat stress. The decrease in chlorophyll concentration due to drought stress indicates that drought is the most damaging stress for chlorophyll concentration. Contrary to this finding, the carotenoid concentration decreased significantly in all applied stresses. The carotenoid concentration of the abiotic stress-affected *V. radiata* seedlings varied slightly between stresses; salt stress caused the greatest reduction in carotenoid concentration (Figure 4). 

The TSP concentration was significantly affected by salt, heat, and drought stress. The most significant reduction in TSP concentration was observed in plants subjected to salt stress, followed by heat and drought stresses (Figure 4). 

The ROS-scavenging enzymes SOD, POD, and catalase represent the most common mechanism used by plants for the detoxification of ROS in abiotic stress conditions [46]. The antioxidant activity (POD and SOD) was significantly reduced under drought, heat, and salt stresses. The activity of CAT decreased after the onset of drought stress in *V. radiata* seedlings, while there was a highly significant increase in response to heat stress and salt stress.

### 2.5. Effect of Abiotic Stress on Na^+^ and K^+^ Concentration in V. radiata

The K^+^ concentration was adversely affected by heat, drought, and salt stress (Table 3). Salt stress caused a highly significant decrease in K^+^ concentration, while other stresses did not cause a significant reduction; still, these stresses resulted in decreased K^+^ concentrations. Conversely, Na^+^ concentration increased in response to all applied stresses; however, only the salt stress caused a significant increase.

### 2.6. Expression of K^+^ Transporters in Drought, Heat, and Salt Stress Conditions

**Figure 5:**
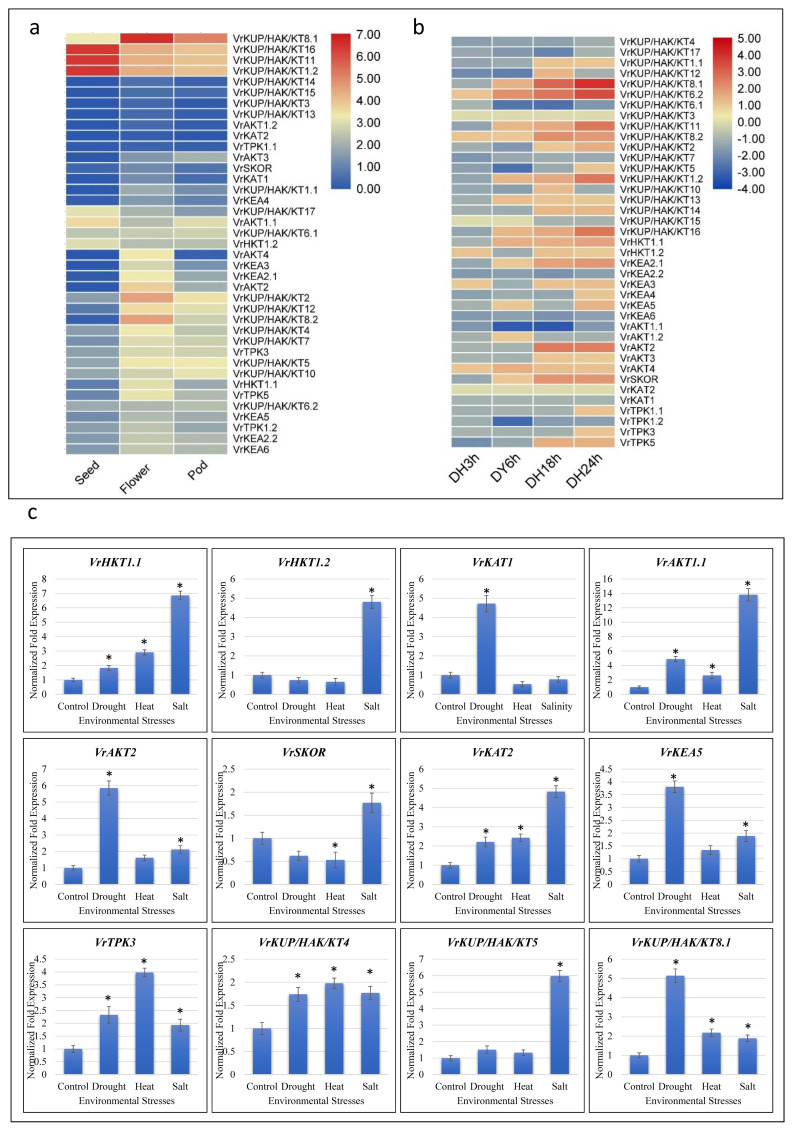
(**a**) Representation of the tissue-specific expression of potassium transport genes in normal conditions. Tissue samples were collected from seeds, pods, and flowers. (**b**) Representation for the expression of potassium transport genes in control and dehydration stress conditions (the blue color represents an absence of a gene, while the red color represents the expression level for highly expressed genes). (**c**) Relative qRT-PCR assay of selected potassium-related genes under heat, drought, salt, and heat stresses. The default expression value for each gene was 1 in non-treated plants. (* *p* < 0.05).

Publicly available RNA-seq data [47] was analyzed to check the expression of potassium transport genes across three different tissues (seed, flower, and pod). It was observed that the expression of 39 genes was detected in normal conditions across three tissues (flower, pod, and seed). The expression of three genes, *VrKUP/HAK/KT1.2*, *VrKUP/HAK/KT11,* and *VrKUP/HAK/KT16* was, relatively, very high in seeds and flowers, while the expression of *VrKUP/HAK/KT8.1* was higher in both flowers and pods. The expression of *VrKUP/HAK/KT8.2* was higher in flowers (Figure 5a). In addition, the expression of *VrAKT4* was slightly higher in flowers and lower in seeds and pods, respectively. These expression patterns indicate the importance of these genes in reproductive tissues. We also checked the expression of the identified transporters and channels in publicly available RNA-seq data [48] for the dehydration stress response. The expression of 28 genes out of 39 was observed in dehydration stress at 24 h (DH24h). The expression of two out of 39 genes (*VrKUP/HAK/KT8.1* and *VrKUP/HAK/KT6.2*) was highly increased at DH18h and DH24h, respectively. Moreover, the expression of *VrSKOR* and *VrAKT2* was also increased at DH18h and DH24h, respectively. Almost no effect was observed on the expression of *VrKUP/HAK/KT3* and *VrKAT2* in all conditions, i.e., DH3h, DH6h, DH18h, and DH24h (Figure 5b). The above results showed that many of these genes were expressed as an immediate response to dehydration stress in seeds. RNA-seq data from two different sources suggest that potassium transport-related genes are not only expressed in different tissues but are also modulated in expression during the stress response.

In order to provide information on the expression profiles of potassium transport-related genes in leaves in response to multiple stresses, we studied the expression of selected genes by real-time RT-qPCR in *V. radiata* leaves. In total, 12 out of 39 genes were selected that are differentially expressed, according to RNA-seq data. In this regard, environmental stresses (drought, heat, and salinity) differentially regulated the expression of *VrHKT1.1, VrHKT1.2, VrKAT1, VrAKT1.1, VrKAT2*, *VrSKOR, VrKEA5, VrAKT2, VrTPK3, VrKUP/HAK/KT4, VrKUP/HAK/KT4,* and *VrKUP/HAK/KT5* (Figure 5c). In response to drought stress, a significant variation of expression was observed for *VrHKT1.1, VrKAT1, VrAKT1.1, VrAKT2, VrKAT2, VrKEA5, VrTPK3, VrKUP/HAK/KT4,* and *VrKUP/HAK/KT8.1.* In response to heat stress, a significant response of expression was observed for *VrHKT1.1*, *VrATK1.1, VrSKOR, VrKAT2, VrTPK3, VrKUP/HAK/KT4,* and *VrKUP/HAK/KT8.1.* Similarly, the expression of *VrHKT1.1, VrHKT1.2, VrAKT1.1, VrAKT2, VrKAT2, VrKEA5, VrTPK3, VrKUP/HAK/KT4*, *VrKUP/HAK/KT5* and *VrKUP/HAK/KT* 8.1 was significantly upregulated in response to salt stress. A significant response of plants under drought stress is the uptake of the solute K^+^ [49,50] to reduce the water potential in cells. Both in drought and osmotic stress, the upregulation of *AKT2* facilitated *O. sativa* with a concomitant increase in growth and the uptake of K^+^ in the root [51]. In *A. thaliana,* first, two Shaker channels, *KAT1* and *AKT,* were identified by the functional complementation of a yeast strain deficient in the uptake of K^+^ [52,53]. Later, in the screening of numerous cDNA libraries, conserved domains encoded by probes between *KAT1* and *AKT1* channels resulted in the identification of three other Shaker channels in *A. thaliana*: *KAT2* [54,55], *AKT2* [56], and *AtKC1* [57]. Therefore, the identification of *SKOR*, *KAT1,* and *AKT1*-like genes in *V. radiata* will help to identify the other members of this family.

## 3. Discussion

### 3.1. K^+^ Transporters and Channels in V. radiata Are Similar to Other Plant Species

The more or less similar number of K^+^ transporters and channels in *V. radiata* (Figure 1) is consistent with previous studies indicating that the studied gene families are evolutionarily conserved in *V. radiata*, other legumes, and non-legume plants, i.e., *A. thaliana*, rice, soybean, wheat, and the common grapevine [10,38,41,43]. This could also indicate the functional-relatedness of the identified K^+^ transporters and channels in *V. radiata* [58]. We posit this because we found multiple sequence features in common in *V. radiata* and other species, e.g., VrKUP/HAK/KT, and those of Arabidopsis shared the same number of TMSs. Similarly, KEA members of both species showed conservation of “G” residues. Previously, it has been established that a divergence in the amino acid sequences of proteins is related to the functional divergence of proteins and vice versa [59]. Among K^+^ channels, the presence of ANK, Ion_trans_2, and KHA domains in the Shaker proteins, similar to Arabidopsis K^+^ channels, suggest their functional similarities [39,60]. Similar to these features, the presence of the highly conserved motifs, i.e., RSXpS/pTXP, GYGD (and EF) motifs, in TPK members is also indicative of possibly similar functional activities in *V. vinifera,* as in Arabidopsis and *O. sativa* [31] (Figure 2).

### 3.2. Abiotic Stress Significantly Affects V. radiata Seedling Growth

Mung beans, like other plants, is greatly affected by climate change and specifically by abiotic stresses. However, efforts to circumvent these stress effects and improve the stress tolerance in mung beans are rare and require special attention [61]. The onset of abiotic stress, i.e., drought, heat, and salt stress, causes a reduction in chlorophyll concentrations in different agricultural plants including legumes [62,63]. The reduction of chlorophyll concentration in the applied stresses in mung bean seedlings is consistent with the earlier reports [62,63,64]. The relatively higher negative effects of drought stress on chlorophyll concentration suggest that water deficiency directly influences the photosynthetic efficiency of mung bean seedlings. We state this because it is known that a reduction in chlorophyll adversely affects photosynthesis [65]. Along with the reduction in chlorophyll concentrations, the applied stresses also significantly affected the carotenoid concentration in mung beans (Figure 4). The concomitant reduction in both chlorophyll and carotenoid concentrations also implies that both are correlated, as reported earlier [66]. This indicates that abiotic stresses can affect pigments other than chlorophyll in mung beans. These results are in accordance with an earlier study, which found that salt and heat stress significantly reduced carotenoid concentrations in *Jatropha* plants [67]. The greater influence of salinity stress on carotenoid concentrations is possibly due to disturbances in the carotenoid biosynthesis pathway, since it has been reported that salt stress reduces the expression of β-carotene synthases [68]. It is known that carotenoids may also function as protectants of photosynthetic apparatus against environmental stresses [69]. Thus, together, the reduction in both carotenoids and chlorophyll significantly disturb the physiology of mung beans at the seedling stage. Other indicators of physiological disturbances in plant health include changes in TSP levels and in the activity of enzymes that help in ROS scavenging [70,71]. The significant decrease in all the studied abiotic stresses is in accordance with the earlier reports. For example, a study on different tomato cultivars that were challenged with salt stress showed a significant reduction in TSPs. Different authors have associated this decrease in TSP with increased proteolysis, a decrease in the availability of amino acids, and the denaturation of enzymes that regulate protein synthesis [72,73]. Thus, in mung bean seedlings, the reduced TSP levels could be due to these reasons when under the applied stresses. In particular, the significantly higher decrease in TSP levels under salt stress is most relevant to earlier reports that salinity affects soluble proteins, mainly due to the loss of the activity of protein-synthesizing enzymes and changes in amino acid concentrations [72]. Under such stresses, plants also struggle to tolerate them by mobilizing several enzymes involved in ROS scavenging and defense responses. SOD alternatively catalyzes the dismutation of the superoxide radical and converts it into O_2_ and H_2_O_2_. Under abiotic stress conditions, the lower SOD activity, as compared to control, is indicative of the lower production of O_2_ and H_2_O_2_ [74]. Thus, it is possible that the mung bean seedlings reduced their SOD activity in order to lower their ROS production. Similarly, the reduced activity of POD under the studied abiotic stresses suggests its involvement in defense in the mung beans against the applied stresses. Finally, the activity of CAT was significantly reduced in the case of drought stress, which is consistent with the results that drought stress decreased the CAT activity in ten *Brassica napus* L. cultivars [75]. On the other hand, the contrasting increase in CAT activity under the influence of heat and salt stress is consistent with the responses of *Acacia retinodes*, *Biota orientalis*, and *Casuarina equisetifolia* when challenged with heat stress [76] and in *Amaranthus tricolor* L. when challenged with salinity stress [77]. These results suggest that CAT activity plays an essential role in mung bean seedlings under salt and heat stresses (Figure 4). 

Overall, our results propose that abiotic stresses, i.e., salt, heat, and drought, significantly affect the physiology of mung bean seedlings, as evident from reduced pigment concentrations and TSP. In response to these stresses, mung bean seedlings activate their defense mechanisms to scavenge the stress-induced effects, e.g., ROS, or the reduced activities of these enzymes indicate that the seedlings have reached a physiological state where the tissues were damaged. Future studies on the recovery of mung bean seedlings after rewatering and on how the activities of these enzymes change will enable us to specify their roles in ROS scavenging and defense responses.

### 3.3. Abiotic Stress Modulates the Expression of K^+^ Transporters and Channels in V. radiata

K^+^ is one of the most abundant cations in plant cells and is involved in plants’ physiological and metabolic processes [78]. The K^+^ concentration in plants is primarily regulated by the influx and efflux of K^+^, with the help of K^+^ transporters and channels [79]. K^+^ transporters and channels have been implicated in plant growth and development ([80] and references therein). Our results also proposed similar roles of K^+^ transporters, since we observed the expression of *VrKUP/HAK/KT1.2, VrKUP/HAK/KT11, VrKUP/HAK/KT16 VrKUP/HAK/KT8.2, and VrAKT4* in different mung bean tissues (Figure 5). Studies have reported that abiotic stresses influence the expression of K^+^ transport-related genes in different plant species, e.g., willow [78], mung bean [81], and wheat [82]. In particular, it is known that salinity stress (and, consequently, a higher Na^+^ concentration in plants) affects K^+^ uptake levels [83]. Conversely, adding K^+^ to plant nutrition reduces the negative effects of salt stress on plants [84]. Our findings, showing that under salt stress the K^+^ concentrations decreased compared to control, are consistent with these observations (Table 3). The expressions of all examined genes except *VrKAT1* were increased under the influence of salt stress in the mung bean seedlings (Figure 5c). The increased expression of these genes suggests that when the Na^+^ concentration increases during salt stress, the K^+^ levels in plant cells reduce and the plant increases the expression of K^+^ transporters and channels for K^+^ homeostasis [58]. Similarly, drought stress also influences the net influx of K^+^ into the plant tissues by modulating (increasing) the expression of related genes, e.g., the expressions of *MdHKT1* and *MdHAK3.2* was increased under drought stress in apple roots [85]. Our results, showing that the expression of *VrHKT1.1, VrKAT1, VrAKT1.1, VrKEA5, VrAKT2, VrTPK3, VrKUP/HAK/KT5, VrKAT2, VrKUP/HAK/KT4, VrKUP/HAK/KT8.1* were increased in mung bean seedlings under the influence of heat and drought stress, are consistent with these reports (Figure 5c). Taken together, it could be proposed that in mung bean seedlings, the onset of drought, heat, and salt stress modulates the expression of K^+^ transport-related genes. Furthermore, the K^+^ transporters and channels are probably involved in mung bean growth and development. 

## 4. Materials and Methods

### 4.1. Data Retrieval and Identification of Potassium Transporters and Channels

Genbank was searched to identify putative K^+^ transport-related genes in *V. radiata*. The protein sequences of respective genes from *O. sativa* and *A. thaliana* [27,30] were used as a query to identify the K^+^ transport-related genes in *V. radiata*. Raw data were manually curated for the elimination of false-positive results. Furthermore, the selective K^+^ filter G-Y-G-D was also manually confirmed in the protein sequences and redundant sequences were removed. In addition, the following motifs were searched in the sequences: two motifs for K^+^ channels (1) A-x-x-T-x-G- [Y, F, L]-G- [D, E], (2) R–[R, Y]- [Y, T]-x-G-Y-G-D; three motifs for HKTs (1) A- [Y, F]-G-x- [V, I]-G- [L, F, Y]- [S, T], (2) G- [I, T]-M-x-S-P-L-Y, (3) T-Y-G- [S-A]-V-G-F-S; and three motifs for KUP/HAK transporters (1) [A, G]- [D, S, G]- [V, L, I, M]-x-x- [S, A]-P-L-Y, (2) [A, G, S]- [D, N]- [G, S, A, C]-x- [L, I, V, F]-x-P-x- [V, I, L, M]- [A, S], (3) [Y, F]-x-x- x-x-x- [H, F, Y]-G-[E-R] -G. The variant of all genes was crosschecked and only large ORFs were used for additional analysis. Conserved domains were further examined using the SMART database (http://smart.embl-heidelberg.de/; accessed 2 January 2021), the NCBI conserved domain database (http://www.ncbi.nlm.nih.gov/Structure/cdd.html) (accessed 2 January 2021), and Pfam database (http://pfam.janelia.org/) (accessed 9 January 2021). All the genomic sequences, the protein length of potassium transporters and channels, chromosomal location, and exon number in each gene were taken from NCBI. The isoelectric point and molecular weight of identified genes were determined using the Expasy tool, Compute PI/MW (https://web.expasy.org/compute_pi/) (accessed 15 January 2021). 

The MEME tool (http://meme.sdsc.edu/meme/meme.html) (accessed 15 January 2021). was used to determine the conserved motifs present in the protein sequences of K^+^ transporters and channels. Default parameters were used for determining the highly conserved motifs. The coding and genomic sequences of all predicted genes were downloaded from NCBI. 

### 4.2. Phylogeny, Gene Structure, Physical Mapping, and Duplication Analyses

ClustalW was used to carry out multiple sequence alignment. The phylogenetic tree was constructed via MEGA7, using the neighbor-joining (NJ) method with replicates of 1000 bootstrap, and visualized using iTOL. Multiple sequence alignment was graphically presented by sequence logos through weblogo3 (http://weblogo.threeplusone.com/) [86] (accessed 8 February 2021). 

Gene structure display server (GSDS) (http://gsds.cbi.pku.edu.cn/) (accessed 15 February 2021) was used in order to construct a schematic representation of the gene structure. Gene duplication events were determined by DNAsp and the Ka/Ks ratio was calculated to determine duplication events. The genomic loci of putative K^+^ channels and transporters were then graphically represented, using the desktop version of Map Chart program (http://www.biometris.wur.nl/UK/Software/MapChart/download) (accessed 12 March 2021) [87].

The promoter sequences (1000 bp upstream ATG region of coding sequence) of selected genes were analyzed to find cis-regulatory elements involved in the regulation of genes in different conditions. The promoter analysis was restricted to a 1000 bp region upstream ATG because most of the important cis-acting elements are found in this region. Moreover, it reduces the occurrence of promoters overlapping with adjacent genes, introns as well as distal promoter regions of other genes [88,89,90]. The plant care database (http://bioinformatics.psb.ugent.be/webtools/plantcare/html/search_CARE.html) was used to predict the cis-regulatory elements [91] (accessed 12 March 2021). 

### 4.3. Plant Material and Stress Imposition

Seeds of *V. radiata* (NIFA Mung17) were collected from AARI (Ayub Agriculture Research Institute, Faisalabad, Pakistan) and were grown in peat moss-filled pots kept under the following growth conditions: temperatures were 22 °C at night and 25 °C by day, with a 16/8 h light/dark period, and 68% humidity. After 12 days of germination, plants were subjected to heat, drought, or salt stress. For heat stress, plants were kept in the incubator at a temperature of 42 °C for 12 h. For drought stress treatment, the water supply was stopped for 8 days (60% field capacity). For salt stress, 10 mL NaCl solution with 100 mM concentration was applied to plants, and tissues were collected after 2 days. The control plants were fully watered throughout the experiment. Three biological replications were conducted for each sample and each replicate included 3–4 plants. The leaf samples of all treated and control plants were directly preserved in liquid nitrogen and kept at −80 °C. 

### 4.4. Physiological and Biochemical Analyses 

#### 4.4.1. Chlorophyll Concentration Measurements

Fresh leaves (100 mg) were ground and homogenized in 80% methanol. These homogenized samples were kept at 4 °C overnight. Subsequently, a spectrophotometer (UH5300, Tokyo, Japan) was used to take the absorbance at three wavelengths, i.e., 480 nm, 645 nm, and 663 nm. An 80% methanol solution was used as a blank to normalize the absorbance value of the solvent. The following formulas were used to measure chlorophyll a and b, total chlorophyll, and chlorophyll a/b ratios [92]:

$$\begin{array}{l} Chlorophyll\;a\left(mg\;g^{-1}f.wt\right)=\left[12.7\;\left(OD\;663\right)-2.69\;(OD\;645)\right]\times V/\left(1000\right)\times W\\ Chlorophyll\;b\left(mg\;g^{-1}f.wt\right)=\left[22.9\;(OD\;645)-4.68\;(OD\;663)\right]\times V/\left(1000\right)\times W\\ Total\;chlorophyll=chlorophyll\;a^{+}chlorophyll\;b\\ Chlorophyll\;a/b\;ratio=Chlorophyll\;a/chlorophyll\;b \end{array}$$

where V = volume of the extract (mL) and W = weight of fresh leaf tissue (g).

#### 4.4.2. Carotenoid Concentrations Measurement

To measure carotenoid concentrations, a weight of 100 mg fresh leaves were taken and homogenized in 80% methanol. Subsequently, samples were centrifuged for 15 min at 12,000 rpm and the supernatant was used to measure the carotenoids concentrations by taking a reading of the absorbance at 480 nm with a spectrophotometer.

#### 4.4.3. Biochemical Studies 

The antioxidant activities of peroxidase (POD), superoxide dismutase (SOD), catalase, and total soluble protein were measured. 

First, fresh leaves (0.5 g) were ground in 1 mL phosphate buffer. Then, 50 µL of the homogenized sample was added to two cuvettes. In one cuvette, 700 µL potassium phosphate buffer, 100 µL guiacol, and 100 µL H_2_O_2_ were added. In the other cuvette, 50 µL nitro blue tetrazolium (NBT), 50 µL riboflavin, 250 µL potassium phosphate buffer, 100 µL methionine, 100 µL triton-X and 400 µL distilled water were added and kept under heavy light for 15 min. Afterward, both cuvettes were gently mixed and the mixture was used to measure POD and SOD activity by recording the absorbances at 470 nm and 560 nm, respectively, with a spectrophotometer [93,94]. For catalase activity measurement, 0.1 mL of enzyme extract was mixed with 1 mL H_2_O_2_ and 2.8 mL phosphate buffer. The absorbance was measured at 240 nm for catalase estimation [95]. Then, 0.5 g of plant leaf material was ground in 10 mL buffer and centrifuged at 11,000 rpm for 10 min at 4 °C. Then, 100 µL aqueous phase with 2 mL Bradford reagent was added to the test tube and kept for 15 min. Afterward, the absorbance was taken at 595 nm under a spectrophotometer for measuring the total soluble proteins [96]. 

#### 4.4.4. Determination of Na^+^ and K^+^ Concentrations 

Plant samples were dried by keeping them in an oven at 80 °C for 48 h. These samples were ground and then treated using an acid digestion method (at 80 °C) with an HClO_4_: HNO_3_ (1:5 *v*/*v*) mixture [97]. The flame spectrophotometer was used to estimate the K^+^ and Na^+^ concentrations in these samples. 

#### 4.4.5. Statistical Analysis 

For statistically significant results, all the experiments were replicated three times. The data has been presented as the average of all the replicates ± SD (standard deviation). To calculate statistical significance among the replicate samples, a two-tailed Student’s *t*-test was performed. A value of *p* < 0.05 was considered significant and *p* < 0.01 was considered to be highly significant. 

### 4.5. In-silico Expression of Potassium Transport Genes in Different Tissues

To analyze the expression pattern of potassium transport genes in different tissues (flowers, pods, and seeds), we downloaded the RNA-seq data (NCBI Bio-project PRJNA276314, experiment run#SRR2177452, SRR2177454 and SRR2182080) from NCBI-SRA (https://www.ncbi.nlm.nih.gov/sra) [47] (accessed 9 June 2021). We also downloaded RNA-seq profiles of the accession numbers SRR3735179, SRR3735193, SRR3735547, SRR3735572, SRR3735589, SRR3735674, SRR3735739, and SRR3735764 [48], to compare the expression pattern of K^+^ transport genes in control conditions, under dehydration stress at different development stages, i.e., 3 h, 6 h, 18 h, and 24 h. An index of the *V. radiata* genome sequence was built using bowtie2 and paired-end clean reads were mapped to the *V. radiata* genome [98]. The expression level of the annotated genes in the reference genome was then calculated using the cufflinks program [99]. The FPKM values were used to construct a heatmap using TBtools [100].

### 4.6. RNA Extraction, cDNA Synthesis, and qRT-PCR Analysis

Total RNA was extracted from the fresh leaves of all treated and control samples with a Thermo Scientific^TM^ GeneJET plant RNA purification kit according to the manufacturer’s instructions and quantified with a Thermo Nanodrop 2000 (Thermo Fisher Scientific, Waltham, MA, USA). One microgram of the RNA sample was used for cDNA synthesis with an All-in-One First-Strand synthesis kit (Thermo Fisher Scientific, Waltham, MA, USA). The cDNA was stored at −20 °C for further use. Gene expression analysis was carried out by qRT-PCR (CFX96 Touch™ Real-Time PCR Detection System) with iTaq Universal SYBR Green SuperMix. An online tool, the “Oligo Calculator” (http://mcb.berkeley.edu/labs/krantz/tools/oligocalc.html) (accessed 10 June 2021) was used to design gene-specific primers, and primer specificity was confirmed by the NCBI primer BLAST (https://www.ncbi.nlm.nih.gov/tools/primer-blast/) (Table S3 (accessed 10 June 2021). The actin gene was used as the housekeeping gene for the normalization of the expression data [47]. A two-tailed Student’s *t*-test was performed for three replicates. A value of *p* < 0.05 was considered to be significant.

## 5. Conclusions

On the basis of structural and sequence identity with known K^+^ transporters, 39 genes were found in *V. radiata,* which were divided into 27 K^+^ transporters and 12 channels. In addition, detailed gene structure analysis and phylogenetic analysis yielded information about conservation in legumes/non-legumes and monocot/dicot plants. Abiotic stresses adversely affect the chlorophyll and carotenoids. The mung bean seedlings showed the changed activities of SOD, POD, and CAT when challenged with salt, heat, and drought stresses. An ionic imbalance was also observed in the mung bean seedlings under the influence of the studied abiotic stresses. Several K^+^ transport genes were expressed in different tissues (seeds, flowers, and pods) and in abiotic stress-responsive manners. Gene expression analysis showed the potential involvement of *VrHKT(1.1* and *1.2), VrKAT (1* and *2) VrAKT1.1, VrAKT2, VrSKOR, VrKEA5, VrTPK3* and *VrKUP/HAK/KT (4, 5,* and *8.1)* in the abiotic response. The present study gives our first insight into K^+^ transporter genes in *V. radiata,* which will be helpful to explore the function of these genes in abiotic stress. 

## Figures and Tables

**Table 1:** Overview of the sequence features of potassium transport genes.

Sr#	Locus Tag	Gene Name	Protein ID	Isoelectric Point	Molecular Weight	TM Domains	Domains	Protein Length	Chr #
**1**	LOC106764953	VrKUP/HAK/KT1.1	XP_014504897.1	6.89	84.60425	13	K_trans	759	6
**2**	LOC106785321	VrKUP/HAK/KT1.2	XP_014501656.1	6.12	91.40354	14	K_trans	791	5
**3**	LOC106762218	VrKUP/HAK/KT2	XP_014501480.1	6.7	88.31495	12	K_trans	791	5
**4**	LOC106753929	VrKUP/HAK/KT3	XP_014491302.1	8.66	87.40831	13	PLN	784	Unknown
**5**	LOC106767047	VrKUP/HAK/KT4	XP_014507349.1	9.28	88.27794	11	K_trans	790	7
**6**	LOC106775695	VrKUP/HAK/KT5	XP_014518335.1	5.79	93.4367	11	PLN	840	10
**7**	LOC106779868	VrKUP/HAK/KT6.1	XP_014523566.1	8.65	85.72046	12	K_trans	772	Unknown
**8**	LOC106779674	VrKUP/HAK/KT6.2	XP_014523333.1	8.66	86.64245	13	PotE, K_trans	776	Unknown
**9**	LOC106773994	VrKUP/HAK/KT7	XP_014516251.1	5.88	94.30594	10	PotE, PLN	846	9
**10**	LOC106771412	VrKUP/HAK/KT8.1	XP_014512874.1	7.83	87.44141	12	PotE, PLN	779	6
**11**	LOC106759282	VrKUP/HAK/KT8.2	XP_014497862.1	7.29	87.06509	13	PotE, PLN	775	4
**12**	LOC106768780	VrKUP/HAK/KT10	XP_014509587.1	8.27	88.70751	13	K_trans, PotE	791	7
**13**	LOC106762322	VrKUP/HAK/KT11	XP_022636150.1	7.59	84.73469	12	K_trans	758	5
**14**	LOC106779912	VrKUP/HAK/KT12	XP_014523613.1	6.63	92.41383	11	PLN, PotE	833	Unknown
**15**	LOC106767048	VrKUP/HAK/KT13	XP_014507351.1	9.44	88.0777	12	K_trans	790	7
**16**	LOC106759816	VrKUP/HAK/KT14	XP_014498681.1	8.2	88.92217	11	K_trans	796	1
**17**	LOC106760231	VrKUP/HAK/KT15	XP_014499185.1	9.02	89.50289	12	K_trans,	804	5
**18**	LOC106760017	VrKUP/HAK/KT16	XP_014498941.1	6.48	68.47955	7	K_trans	610	5
**19**	LOC106766977	VrKUP/HAK/KT17	XP_014507267.1	8.17	80.93343	12	PotE, K_trans	723	7
**20**	LOC106756241	VrHKT1.1	XP_014494069.1	9.39	57.12935	9	TrkH	507	1
**21**	LOC106763952	VrHKT1.2	XP_014503627.1	9.39	58.81727	9	TrkH	518	1
**22**	LOC106774167	VrKEA2.1	XP_014516532.1	4.96	78.72034	10	TrkA_N, RILP	1195	9
**23**	LOC106764844	VrKEA2.2	XP_022638221.1	4.55	56.79963	6	Na_H_Exchanger	527	7
**24**	LOC106768449	VrKEA3	XP_014509103.1	5.52	85.86885	1	KefB, TrkA_N	790	7
**25**	LOC106769002	VrKEA4	XP_014509921.1	5.77	62.90096	12	Na_H_Exchanger	586	7
**26**	LOC106771900	VrKEA5	XP_014513420.1	6.17	62.48549	11	Na_H_Exchanger	576	1
**27**	LOC107634854	VrKEA6	XP_022638220.1	5.55	56.79963	11	Na_H_Exchanger	595	6
**28**	LOC106766559	VrTPK1.1	XP_014506766.1	5.5	38.65153	5	Ion_trans_2	344	7
**29**	LOC106756712	VrTPK1.2	XP_014494736.1	9.01	43.24297	5	Ion_trans_2	389	3
**30**	LOC106752883	VrTPK3	XP_014490151.1	8.76	47.3163	5	Ion_trans_2, EF-hand_7	425	Unknown
**31**	LOC106764594	VrTPK5	XP_014504362.1	6.11	38.82113	5	Ion_trans_2, EFh	348	6
**32**	LOC106765548	VrAKT1.1	XP_014505698.1	7.04	97.42988	5	ANK, KHA, PLN03192, Ion_trans_2	869	7
**33**	LOC106763693	VrAKT1.2	XP_014503345.1	6.36	99.25835	5	PLN03192, CAP_ED, ANK, Ion_trans_2	875	6
**34**	LOC106776053	VrAKT2	XP_014518839.1	6.2	95.02182	7	PLN03192, CAP_ED, KHA, ANK	832	10
**35**	LOC106775235	VrAKT3	XP_014517810.1	6.51	89.20001	5	PLN03192, Ion_trans_2, KHA	776	10
**36**	LOC106752947	VrAKT4	XP_014490211.1	6.84	83.63201	6	PLN03192, KHA, Ion_trans, CAP_ED	717	Unknown
**37**	LOC106775884	VrKAT1	XP_014518605.1	6.27	89.45222	5	PLN03192, Ion_trans, cNMP_binding, KHA	778	10
**38**	LOC106761761	VrKAT2	XP_022636536.1	8.95	71.49999	5	PLN03192, Ank_2, KHA	623	5
**39**	LOC106765054	VrSKOR	XP_014505028.1	6.46	97.56075	5	PLN03192, ANK, KHA, Ion_trans_2	851	6

Note: transporters (VrKUP/HAK/KT, HKT, KEA), channels (TPK, AKT, KAT, SKOR).

**Table 2:** Promoter analysis of selected potassium channels and transporters.

Regulatory Element	Core Sequence	VrAKT1.1	VrAKT1.2	VrAKT1.3	VrAKT2	VrKAT1.1	VrKAT1.2	VrKAT1	VrSKOR	VrHKT1.1	KT1.2	Function
**ABRE**	CACGTG	1			1			1	1			Response to abscisic acid signals
	ACGTG	1			2	1		3	3		1	
**MYB**	TAACCA		2	4	2	4	2	2	2			Response to drought stress and ABA signals
	CAACCA		1	1	1		2	1	2	2	1	
**MYC**	CATTTG		4	2	2	6	2	3	4	2	3	Response to drought, ABA, and cold signals
**W-box**	TTGACC			1				1	2			Response to SA, GA, and pathogenesis signals
**GT-1 motif**	GGTTAA	1	1	1	3	4	1	1	4			Light-responsive element
**G-box**	CACGTG	1			3			3	3	1		Involved in the light response
**GARE**	TCTGTTG							1				Gibberellin-responsive element
**MBS**	CAACTG	3		7		1	2	4		8		Involved in drought-inducibility
**ARE**	AAACCA		1	1	1	1			4	1		Essential for the anaerobic induction
**TCA-element**	CCATCTTTTT	2	1	1						1		Response to salicylic acid
**TC-rich repeats**	ATTCTCTAAC		2	2		4		10	5		2	Involved in defense and the stress response
**P-box**	CCTTTTG	1									1	Gibberellin-responsive element
**LTR**	CCGAAA		4					2	1			Response to low temperature
**I-Box**	GATAA	10	4	4	10	6	4	3		4	1	Response to SA, GA, and pathogenesis signals
**W-Box**	TGAC	18	12	10	12	10		3	7	9	6	Response to drought, ABA, and cold signals

**Table 3:** Effect of salt, heat and drought stress on Na^+^ and K^+^ concentrations.

Treatments	Na^+^ Concentration (mg g^−1^)	K^+^ Concentration (mg g^−1^)
Control	38.97 ± 1.29	60.74 ± 2.27
Salt	51.71 ± 1.49 **	45.70 ± 1.82 **
Drought	40.21 ± 1.39	56.16 ± 1.41
Heat	39.76 ± 1.12	55.270 ± 1.38

** Represents highly significant differences of mean values at *p* < 0.01. The “±” represents standard deviation from mean values.

## Data Availability

All the datasets included in this study have been presented within the manuscript and/or as supplementary files.

## Supplementary Materials

The following are available online at https://www.mdpi.com/article/10.3390/plants11010002/s1, Figure S1. Conserved exons in lengths among VrKUP/HAK/KT, VrHKT, VrKEA, VrSKOR, VrTPK, and VrKAT families were shown by multiple colors; Figure S2. Structural analysis of potassium channels and transporters genes. Exons and introns of potassium transport-related genes are represented by yellow boxes and black lines, respectively. Gene models are based on CDC Frontier genome Cav1.0 gene annotations; Table S1. Cis-regulatory elements present at the upstream region of potassium channels genes are given; Table S2. Cis-regulatory elements present at the upstream region of potassium transporters genes are given; Table S3: List of primers used for qRT-PCR analysis; Table S4: Description of various conserved domains found in the protein sequences of potassium transport-related proteins.
