# Supplementary material for: Genome-Wide Identification and Expression Profiling of Potassium Transport-Related Genes in Vigna radiata under Abiotic Stresses
Source: Plants (Basel). 2021 Dec 21;11(1):2. doi: 10.3390/plants11010002 (PMC8747342; doi:10.3390/plants11010002)
Supplement: Supplementary file 1 [file plants-11-00002-s001.zip › plants-1356822-supplementary.pdf]

VrKUP/HAK/KT4	Exon 1 140	Exon 2 244	Exon 3 249	Exon 4 51	Exon 5 261	Exon 6 53	Exon 7 255	Exon 8 160	Exon 9 645				
VrKUP/HAK/KT17	Exon 1 258	Exon 2 226	Exon 3 222	Exon 4 51	Exon 5 261	Exon 6 53	Exon 7 220	Exon 8 145	Exon 9 125	Exon 10 152	Exon 11 112	Exon 12 160	Exon 13 60
VrKUP/HAK/KT1.1	Exon 1 258	Exon 2 244	Exon 3 246	Exon 4 51	Exon 5 261	Exon 6 53	Exon 7 87	Exon 8 562					
VrKUP/HAK/KT1.2	Exon 2 493	Exon 1 559	Exon 3 246	Exon 4 51	Exon 5 261	Exon 6 124	Exon 7 56	Exon 8 97	Exon 9 124				
VrKUP/HAK/KT8.1	Exon 1 216	Exon 2 159	Exon 3 226	Exon 4 51	Exon 5 261	Exon 6 261	Exon 7 168	Exon 8 265					
VrKUP/HAK/KT6.2	Exon 1 286	Exon 2 156	Exon 3 249	Exon 4 51	Exon 5 261	Exon 6 54	Exon 7 192	Exon 8 954					
VrKUP/HAK/KT6.1	Exon 1 300	Exon 2 116	Exon 3 157	Exon 4 51	Exon 5 261	Exon 6 54	Exon 7 251	Exon 8 1034					
VrKUP/HAK/KT3	Exon 1 505	Exon 2 229	Exon 3 157	Exon 4 51	Exon 5 261	Exon 6 54	Exon 7 251	Exon 8 53	Exon 9 118				
VrKUP/HAK/KT11	Exon 1 246	Exon 2 275	Exon 3 226	Exon 4 51	Exon 5 261	Exon 6 55	Exon 7 122	Exon 8 255	Exon 9 353	Exon 10 245	Exon 11 118		
VrKUP/HAK/KT8.2	Exon 1 505	Exon 2 229	Exon 3 226	Exon 4 51	Exon 5 261	Exon 6 52	Exon 7 129	Exon 8 234	Exon 9 1018				
VrKUP/HAK/KT2	Exon 1 505	Exon 2 229	Exon 3 224	Exon 4 51	Exon 5 261	Exon 6 54	Exon 7 115	Exon 8 255	Exon 9 496	Exon 10 345	Exon 11 287		
VrKUP/HAK/KT7	Exon 1 587	Exon 2 319	Exon 3 226	Exon 4 51	Exon 5 261	Exon 6 246	Exon 7 168	Exon 8 255	Exon 9 1168	Exon 10 1219			
VrKUP/HAK/KT5	Exon 1 587	Exon 2 319	Exon 3 226	Exon 4 51	Exon 5 261	Exon 6 246	Exon 7 168	Exon 8 255	Exon 9 1168				
VrKUP/HAK/KT1.2	Exon 1 505	Exon2 229	Exon 3 250	Exon 4 51	Exon 5 261	Exon 6 107	Exon 7 115	Exon 8 255	Exon 9 145				
VrKUP/HAK/KT10	Exon 1 587	Exon 2 319	Exon 3 226	Exon 4 51	Exon 5 261	Exon 6 53	Exon 7 114	Exon 8 243	Exon 9 118				
VrKUP/HAK/KT13	Exon 1 218	Exon 2 118	Exon 3 226	Exon 4 51	Exon 5 261	Exon 6 55	Exon 7 216	Exon 8 128	Exon 9 238				
VrKUP/HAK/KT14	Exon 1 140	Exon 2 231	Exon 3 265	Exon 4 51	Exon 5 261	Exon 6 51	Exon 7 193	Exon 8 138	Exon 9 137				
VrKUP/HAK/KT15	Exon 1 300	Exon 2 156	Exon 3 275	Exon 4 51	Exon 5 261	Exon 6 52	Exon 7 163	Exon 8 144	Exon 9 122				
VrKUP/HAK/KT16	Exon 1 244	Exon 2 159	Exon 3 250	Exon 4 51	Exon 5 261	Exon 6 246	Exon 7 112						
VrHKT1.1	Exon 1 1346	Exon 2 202	Exon 3 599										
VrHKT1.2	Exon 3 338	Exon 2 202	Exon 1 1218										
VrKEA2.1	Exon 1 248	Exon 2 1668	Exon 3 172	Exon 4 191	Exon 5 62	Exon 6 53	Exon 7 60	Exon 8 120	Exon 9 112	Exon 10 118	Exon 11 118		
VrKEA2.2	Exon 1 510	Exon 2 44	Exon 3 59	Exon 4 64	Exon 5 62								
VrKEA3	Exon 1 196	Exon 2 203	Exon 3 408	Exon 4 369	Exon 5 62	Exon 6 81	Exon 7 102	Exon 8 78	Exon 9 822	Exon 10 112	Exon 11 76	Exon 12 53	
VrKEA4	Exon 1 592	Exon 2 44	Exon 3 59	Exon 4 79	Exon 5 62	Exon 6 76	Exon 7 72	Exon 8 84	Exon 9 118	Exon 10 58	Exon 11 67	Exon 12 134	
VrKEA5	Exon 1 474	Exon 2 44	Exon 3 78	Exon 4 118	Exon 5 62	Exon 6 76	Exon 7 72	Exon 8 84	Exon 9 69	Exon 9 118	Exon 11 110		
VrKEA6	Exon 1 501	Exon 2 44	Exon 3 64	Exon 4 76	Exon 5 62	Exon 6 72	Exon 7 84	Exon 8 54	Exon 9 118	Exon 10 92	Exon 11 76	Exon 12 53	
VrAKT1.1	Exon 1 338	Exon 2 366	Exon 3 58	Exon 4 318	Exon 5 249	Exon 6 99	Exon 7 24	Exon 8 199	Exon 9 53	Exon 10 553			
VrAKT1.2	Exon 1 310	Exon 2 366	Exon 3 52	Exon 4 322	Exon 5 249	Exon 6 99	Exon 7 24	Exon 8 78	Exon 9 53	Exon 10 67			
VrAKT2	Exon 1 620	Exon 2 219	Exon 3 159	Exon 4 58	Exon 5 249	Exon 6 240	Exon 7 318	Exon 8 76	Exon 9 55				
VrAKT3	Exon 1 748	Exon 2 216	Exon 3 156	Exon 4 58	Exon 5 249	Exon 6 240	Exon 7 99	Exon 8 309	Exon 9 53				
VrAKT4	Exon 1 430	Exon 2 156	Exon 3 58	Exon 4 317	Exon 5 240	Exon 6 249	Exon 7 48	Exon 9 309	Exon 10 253				
VrTPK1.1	Exon 1 1050	Exon 2 253	Exon 3 154	Exon 4 49									
VrTPK1.2	Exon 1 1050	Exon 2 606											
VrTPK3	Exon 1 1050	Exon 2 719											
VrTPK5	Exon 1 104	Exon 2 1050	Exon 3 389	Exon 4 154	Exon 5 67	Exon 6 54	Exon 7 152	Exon 8 51	Exon 9 112	Exon 10 44			
VrKAT1	Exon 1 786	Exon 2 216	Exon 3 156	Exon 4 58	Exon 5 38	Exon 6 240	Exon 7 99						
VrKAT2	Exon 1 279	Exon 2 173	Exon 3 219	Exon 4 163	Exon 5 58	Exon 6 318	Exon 7 246	Exon 8 99					

Supplementary Figure S1. Conserved exons (in lengths) among VrKUP/HAK/KT, VrHKT, VrKEA, Shaker, and VrTPK families were shown by multiple colors.

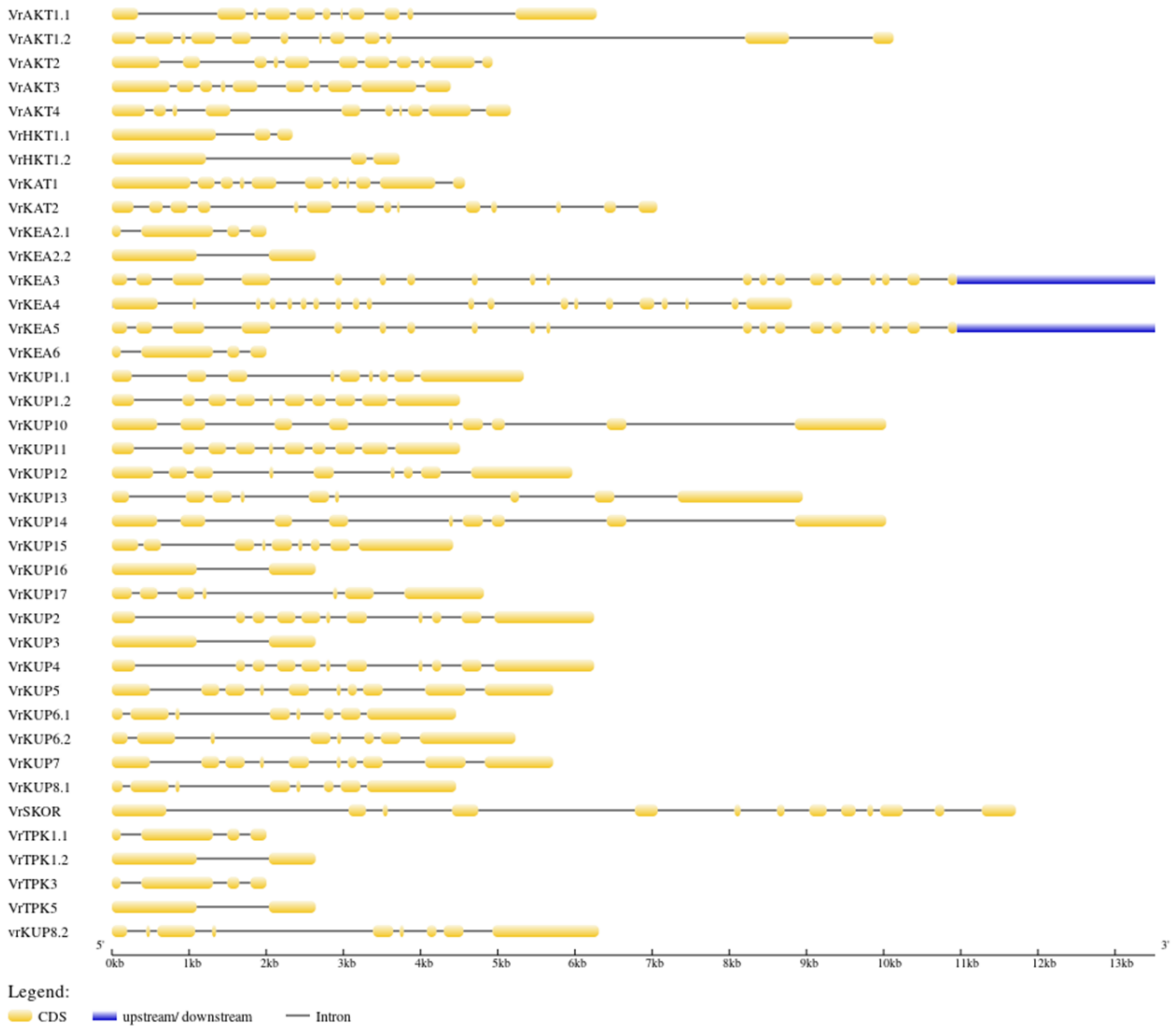

Supplementary Figure S2. Structural analysis of potassium channels and transporters genes. Exons and introns of potassium transport-related genes are represented by yellow boxes and black lines respectively. Gene models are based on CDC Frontier genome Cav1.0 gene annotations.

Supplementary Table S1: *Cis*- regulatory elements present at the upstream region of potassium channel genes are given.

**VrAKT1.1**	AAGAA-motif, ABRE, ACE, ATCT-motif, Box II, CAAT-box, CCAAT-box, G-Box, GA-motif, GATA-motif, GT1-motif, HSE, I-box MSA-like Skn-1_motif, box II, rbcS-CMA7a
**VrAKT1.2**	AAGAA-motif, ABRE, AATCT-motif, Box II, CAAT-box, CCAAT-box, G-box, GA-motif, GATA-motif, GT1-motif, HSE I-box, MSA-like, Skn-1_motif, TATA-box, box II, rbcS-CMA7a
**VrAKT2**	AAGAA-motif, ABRE, ACE, ATCT-motif, Box II, CAAT-box, CCAAT-box G-box, GATA-motif, GT1-motif, HSE I-box, MSA-l, Skn-1_motif, TATA-box, box II, rbcS-CMA7a
**VrAKT3**	AAGAA-motif, ABRE, ACE, ATCT-motif, Box II, CAAT-box, CCAAT-box, G-box, GA-motif, GATA-motif, GT1-motif, HSE, I-box, MSA-like, Skn-1_motif, box II, rbcS-CMA7a
**VrKAT3**	AAGAA-motif, ABRE, ACE, ATCT-motif, Box II, CAAT-box, CCAAT-box, G-Box, GA-motif, GATA-motif, GT1-motif, HSE I-box, MSA-like, Skn-1_motif, TATA-box, box II, rbcS-CMA7a
**VrSKOR**	AAGAA-motif, ABRE, ACE, ATCT-motif, Box II, CAAT-box, CCAAT-box, G-box, GA-motif, GATA-motif, GT1-motif, HSE I-box, MSA-like, Skn-1_motif, TATA-box, box II, rbcS-CMA7a
**VrAKT4**	AAGAA-motif, ABRE, ACE, ATCT-motif, Box II, CAAT-box, CCAAT-box, G-Box, GA-motif, GATA-motif, GT1-motif, HSE I-box, MSA-like, Skn-1_motif, TATA-box, box II, rbcS-CMA7a
**VrTPK5**	AAGAA-motif, ABRE, ACE, ATCT-motif, Box II, CAAT-box, CCAAT-box, G-Box, GA-motif, GATA-motif, GT1-motif, HSE I-box, MSA-like, Skn-1_motif, TATA-box, box II, rbcS-CMA7a
**VrTPK1.1**<br><br>**VrTPK1.2**	AAGAA-motif, ABRE, ACE, ATCT-motif, CAAT-box, CCAAT-box, G-box, GA-motif, GATA-motif, GT1-motif, HSE I-box, MSA-like, Skn-1_motif, TATA-box, box II, rbcS-CMA7a,<br>AAGAA-motif<br>ABRE, ACE, ATCT-motif, Box II, CAAT-box, CCAAT-box, G-Box, GA-motif, GATA-motif, GT1-motif, HSE, I-box, MSA-like, Skn-1_motif, TATA-box, box II, rbcS-CMA7a
**VrTPK3**	AAGAA-motif, ABRE, ACE, ATCT-motif, Box II, CAAT-box, CCAAT-box, G-Box, GA-motif, GATA-motif, GT1-motif, HSE I-box, MSA-like, Skn-1_motif, TATA-box, box II, rbcS-CMA7a
**VrKAT1**	AAGAA-motif, ABRE, ACE, ATCT-motif, Box II, CAAT-box, CCAAT-box, G-Box, G-box, GA-motif, GATA-motif, GT1-motif, HSE, I-box, MSA-like, Skn-1_motif, TATA-box, box II,rbcS-CMA7a

Supplementary Table S2: *Cis*-regulatory elements present at the upstream region of potassium transporters genes are given.

**VrKUP/HAK/KT4**	AAGAA-motif, ABRE, ACE, ATCT-motif, Box II, CAAT-box CCAAT-box G-Box GA-motif GATA-motif GT1-motif HSE I-box MSA-like Skn-1_motif TATA-box box II rbcS-CMA7a
**VrKUP/HAK/KT17**	AAGAA-motif ABRE ACE ATCT-motif Box II CAAT-box CCAAT-box G-box GA-motif GATA-motif GT1-motif HSE I-box MSA-like Skn-1_motif TATA-box box II rbcS-CMA7a
**VrHKT1.1**	AAGAA-motif ABRE ACE ATCT-motif Box II CAAT-box CCAAT-box G-Box GA-motif GATA-motif GT1-motif HSE I-box MSA-like Skn-1_motif TATA-box box II
**VrKUP/HAK/KT1.1**	AAGAA-motif ABRE ACE ATCT-motif Box II CAAT-box CCAAT-box G-Box GA-motif GATA-motif GT1-motif HSE I-box MSA-like Skn-1_motif TATA-box box II rbcS-CMA7a
**VrKUP/HAK/KT12**	AAGAA-motif ABRE ACE ATCT-motif Box II CAAT-box CCAAT-box G-box GA-motif GATA-motif GT1-motif HSE I-box MSA-like Skn-1_motif TATA-box box II rbcS-CMA7a
**VrHKT1.2**	AAGAA-motif ABRE ACE ATCT-motif Box II CAAT-box CCAAT-box G-box GA-motif GATA-motif GT1-motif HSE I-box MSA-like Skn-1_motif TATA-box box II rbcS-CMA7a
**VrKUP/HAK/KT8.1**	AAGAA-motif ABRE ACE ATCT-motif Box II CAAT-box CCAAT-box G-box GA-motif GATA-motif GT1-motif HSE I-box MSA-like Skn-1_motif TATA-box box II rbcS-CMA7a
**VrKUP/HAK/KT6.2**	AAGAA-motif ABRE ACE ATCT-motif Box II CAAT-box CCAAT-box G-box GA-motif GATA-motif GT1-motif HSE I-box MSA-like Skn-1_motif TATA-box box II rbcS-CMA7a
**VrKUP/HAK/KT6.1**	AAGAA-motif ABRE ACE ATCT-motif Box II CAAT-box CCAAT-box G-Box GA-motif GATA-motif GT1-motif HSE I-box MSA-like Skn-1_motif TATA-box box II rbcS-CMA7a
**VrKUP/HAK/KT11**	AAGAA-motif ABRE ACE ATCT-motif Box II CAAT-box CCAAT-box G-Box GA-motif GATA-motif GT1-motif HSE I-box MSA-like Skn-1_motif TATA-box box II rbcS-CMA7a
**VrKUP/HAK/KT7**	AAGAA-motif ABRE ACE ATCT-motif Box II CAAT-box CCAAT-box G-box GA-motif GATA-motif GT1-motif HSE I-box MSA-like Skn-1_motif TATA-box box II rbcS-CMA7a
**VrKUP/HAK/KT3**	AAGAA-motif ABRE ACE ATCT-motif Box II CAAT-box CCAAT-box G-Box GA-motif GATA-motif GT1-motif HSE I-box MSA-like Skn-1_motif TATA-box box II rbcS-CMA7a
**VrKAE6**	AAGAA-motif ABRE ACE ATCT-motif Box II CAAT-box CCAAT-box G-Box GA-motif GATA-motif GT1-motif HSE I-box MSA-like Skn-1_motif TATA-box box II rbcS-CMA7a
**VrKAE3**	AAGAA-motif ABRE ACE ATCT-motif Box II CAAT-box CCAAT-box G-Box GA-motif GATA-motif GT1-motif HSE I-box MSA-like Skn-1_motif TATA-box box II rbcS-CMA7a
**VrKUP/HAK/KT1.2**	AAGAA-motif, ABRE, ACE, ATCT-motif, Box II, CAAT-box, CCAAT-box, G-Box, GA-motif, GATA-motif, GT1-motif, HSE, I-box, MSA-like, Skn-1_motif, TATA-box, box II, rbcS-CMA7a
**VrKEA2.1**	AAGAA-motif, ABRE, ACE, ATCT-motif, Box II, CAAT-box, CCAAT-box, G-box, GA-motif, GATA-motif, GT1-motif, HSE, I-box, MSA-like, Skn-1_motif, TATA-box, box II, rbcS-CMA7a
**VrKEA2.2**	AAGAA-motif, ABRE, ACE, ATCT-motif, Box II, CAAT-box, CCAAT-box, G-Box, GA-motif, GATA-motif, GT1-motif, I-box, MSA-like, Skn-1_motif, TATA-box, box II, rbcS-CMA7a
**VrKAE5**	AAGAA-motif ABRE ACE ATCT-motif Box II CAAT-box CCAAT-box G-Box GA-motif GATA-motif GT1-motif HSE I-box MSA-like Skn-1_motif TATA-box box II rbcS-CMA7a

Supplementary Table S3: List of primers used for qRT-PCR analysis

Sr. No	Gene Name	Primer Sequence
1	**VrHKT1.1F**	CTTTGTCCCCACCAACGAGA
	**VrHKT1.1R**	CACGGTGGATATAGGGCGTT
2	**VrHKT1.2F**	CCTTCCATGTCCACCCCTTC
	**VrHKT1.2R**	GTCTAACGGGGGTTCTTGGG
3	**VrKAT1F**	ACAGGGAAGCCACGGAAAAT
	**VrKAT1R**	AATTGCTGCTCCTACGCCTT
4	**VrAKT1.1F**	ACTAGTGAGCCAACCATGCC
	**VrAKT1.1R**	ACCTTTTCCAGATGGCCCTG
5	**VrAKT2F**	ACTGCAATGTGGGGTGCTAT
	**VrAKT2R**	TCACCTGCTGTGTTCTGGTC
6	**VrSKORF**	ACACTGAGGCTGCTGTCATT
	**VrSKORR**	GGGTGACATCCATAATTGACCA
7	**VrKAT2F**	TTGTGGCAGTCTGTCGTCTG
	**VrKAT2R**	GGTAAACGTTGCATTGGCTGA
8	**VrKEA5F**	ATGGGAAAACAAAACCTTCTGG
	**VrKEA5R**	CAATTTGACGTCGCCCGAAT
9	**VrTPK3F**	AAGTTGCCAAACCCCTCACA
	**VrTPK3R**	GGCTTCTTGAGCCTTCCCAT
10	**VrKUP/HAK/KT4F**	CTGGAGTCCCTGCCAGTTTT
	**VrKUP/HAK/KT4R**	GCACCGGTACAGTCTTCACA
11	**VrKUP/HAK/KT5F**	TTGTCTGAGTGTGGACAGGT
	**VrKUP/HAK/KT5R**	GCAGCTGCTCAAAAGTCTGG
12	**VrKUP/HAK/KT8.1F**	TGTTTGCTTTAACAGGCTTGG
	**VrKUP/HAK/KT8.1R**	GCAAATTGCCAGCAAAATGCTT
13	**VractinF**	TTCCTTTTCTTCGTTTTTCATGCT
	**VractinR**	AAACCGAGGCACCTAATTCTTG

Supplementary Table S4: Description of various conserved domains found in the protein sequences of potassium transport related proteins.

Name of domain	Full name	Accession number	Function
K_trans	*K+ potassium transporter*	PF02705	Potassium transport
PLN	Potassium transporter	PLN00151	Potassium transport
PotE	Amino acid transporter	COG0531	Amino acid transport and metabolism
TrkH	Cation transport protein	cl17365	Active sodium up-take utilizing ATP in the process.
TrkA_N	Potassium ion transmembrane transporter activity	pfam02254	This domain binds to NAD
RILP-like	Rab interacting lysosomal protein-like	cl23720	Regulation of cellular morphology
Na_H_Exchanger	Sodium/hydrogen exchanger family	pfam00999	Maintaining the pH of actively metabolising cells
KefB	Kef-type K+ transport system	COG0475	Membrane component KefB [Inorganic ion transport and metabolism]
Ion_trans_2	Ion channel	pfam07885	This family includes the two-membrane helix type ion channels found in bacteria.
EF-hand_7	EF-hand domain pair	pfam13499	Calcium-binding proteins.
EFh	EF-hand	cl08302	Calcium binding motif
ANK	ANK repeat	sd00045	Scaffolds for protein-protein interactions
KHA	Kha	pfam11834	Dimerization domain of potassium ion channel
CAP_ED	Effector domain of the CAP family of transcription factors	cd00038	Binds cAMP, FNR (fumarate and nitrate reduction), which uses an iron-sulfur cluster to sense oxygen) and CooA, a heme containing CO sensor
cNMP_binding	Cyclic nucleotide-binding domain	PF00027	Cyclic nucleotide-binding
PLN03192	Voltage-dependent potassium channel	PLN03192	Voltage-dependent potassium transport.
